# Neuromusculoskeletal Modeling and Force Prediction: Verification Through Experimental Neuromuscular Dynamics

**DOI:** 10.1007/s10439-025-03783-2

**Published:** 2025-07-08

**Authors:** Colton D. Babcock, Landon D. Hamilton, Anastasios Lykidis, Richard Babcock, Ioannis G. Amiridis, Clare K. Fitzpatrick

**Affiliations:** 1 Mechanical and Biomedical Engineering, Boise State University, 1910 University Drive, MS-2085, Boise, ID 83725-2085 USA; 2 UC Health, Medical Center of the Rockies, Loveland, CO USA; 3 Laboratory of Neuromechanics, Department of Physical Education and Sports Sciences at Serres, Aristotle University of Thessaloniki, Serres, Greece

**Keywords:** High-density electromyography, Musculoskeletal modeling, Finite element, Neural modeling

## Abstract

**Purpose:**

Neuromusculoskeletal (NMS) function is influenced by the interactions between neural and musculoskeletal systems. Age-related changes in motor unit morphology contribute to changes in motor control and force production with advancing age; however, a better understanding of the underlying mechanisms between force production and motor unit reorganization and their interrelationships is needed to develop targeted therapies and interventions to age-related changes. Direct experimental measurement of these neuromuscular changes is challenging due to ethical and logistical constraints and the complexity of isolating individual motor unit contributions in vivo, particularly across time. Computational modeling provides a complementary approach which can help bridge this gap. The objective of this study is to develop a computational framework for predicting dorsiflexion force profiles through the translation of experimental motor unit recordings into simulated musculoskeletal responses.

**Methods:**

This study presents the development of a combined NMS model that integrates experimental motor unit recordings into a musculoskeletal simulation framework. Specifically, the NMS model predicts dorsiflexion force profiles by translating experimental data from high-density electromyography recordings into simulated subject-specific motor unit discharge characteristics and simulated muscle responses. The NMS model incorporates a detailed motor neuron pool simulation and a finite element musculoskeletal model, allowing for physiologically accurate representation of motor unit discharge characteristics, muscle force generation, and force variability.

**Results:**

The accuracy of the simulated force profiles in predicting the experimental force were 10.25 N and 0.95, respectively, for average root mean square error and R^2^ values. Results demonstrate strong agreement between simulated and experimental force profiles and motor unit recordings.

**Conclusion:**

By bridging the gap between computational and experimental approaches, this study aims to enhance understanding of NMS dynamics and support the development of personalized treatment strategies for neurodegenerative disease patients.

## Introduction

Neuromusculoskeletal (NMS) function involves complex interplay between the nervous and musculoskeletal systems. During healthy aging, as well as neurodegenerative and neurodevelopment impairment, biological parameters are altered at multiscale levels – for example, age-related changes in motor unit morphology can lead to slower contractile velocity and increased fatigability resulting in decreased force steadiness during motor tasks [1]. However, these parameters are often inherently difficult to measure in vivo during experimental or clinical studies due to ethical considerations and logistical constraints [2, 3]. Improved insight into these changes, and interaction between neural and musculoskeletal parameters, could offer vital insights for aging and neurodegenerative research. With the growing prevalence of neurodegenerative diseases, including Alzheimer’s disease, Parkinson’s disease, and amyotrophic lateral sclerosis (ALS) [4, 5], there is increasing need to better understand these conditions to optimize healthy aging and life-long function and mobility. Alternative approaches and tools to assist in understanding NMS function may ultimately play a role in improving neuromusculoskeletal health across the lifespan. This has led to a growing interest in developing innovative, non-invasive platforms that can complement traditional clinical research methodologies [6–8].

In this context, computational modeling, particularly multiscale NMS models, has emerged as a promising tool to supplement experimental research and help bridge the gap to clinical application. The utility of neuromuscular models has been particularly evident in the study of motor unit (MU) recruitment and discharge behavior, areas that are difficult to observe directly in vivo [9–12]. Recent advances in neuromuscular modeling have provided valuable insights into common synaptic input (CSI) and its role in coordinating motor neuron activity [13–15]. These models incorporate detailed representations of motor neuron electrophysiological properties and spinal synaptic inputs, often derived from high-density electromyography (HD-EMG) recordings. Validation of these models against experimental data has confirmed their ability to predict motor neuron discharge patterns [14], making them a potential tool for studying neuromuscular function. Similarly, musculoskeletal models have long been a cornerstone of biomechanics research, offering a means to analyze complex geometry, deformable materials, and kinematic interactions within the human body [16–20]. Platforms such as OpenSim have enabled the simulation of EMG-driven rigid body kinematics and the analysis of pre- and post-surgical gait alterations [21–23]. However, rigid body models fall short when investigating deformable metrics like stress and strain within muscles. In contrast, finite element (FE) modeling is particularly well suited for solving problems related to continuum elasticity and structural analysis, making it an ideal approach for simulating musculoskeletal systems in a more detailed and anatomically accurate manner [24, 25].

Recent advances in FE modeling have allowed for the development of subject-specific musculoskeletal models that incorporate detailed anatomical geometry derived from medical imaging. These models commonly simulate muscle tissue as a transversely isotropic material, accounting for both simplified and advanced fiber orientations [26–31]. The increased anatomic and material detail offered by FE models makes them an excellent complement to neuromuscular simulations, particularly when addressing research questions related to muscle stress and strain, which are useful metrics in analyzing muscle injury and force generation, as well as contraction dynamics which is an important aspect of recreating musculoskeletal behavior in silico.

Given the growing recognition of NMS models in neurodegenerative disease research, we will build a combined NMS model that can account for both neural and musculoskeletal dynamics and incorporates subject-specific neuromuscular metrics. The objective of this study is to develop a computational framework for predicting dorsiflexion force profiles through the translation of experimental motor unit recordings into simulated musculoskeletal responses. We will validate our approach against experimentally collected HD-EMG data through comparison of key neuromuscular metrics, including interspike interval (ISI), standard deviation (STD) of the cumulative spike train (CST), and the coefficient of variation (CoV) of the ISI. The integration of a detailed motor neuron pool simulation with an anatomically accurate FE musculoskeletal model offers a novel approach to exploring participant-specific neuromuscular dynamics in a controlled, virtual environment. With direct experimental measurement of neuromuscular changes being challenging due to ethical and logistical constraints, as well as the complexity of isolating individual motor unit contributions in vivo, computational modeling is a key analysis tool to overcoming these limitations by providing a framework to investigate neuromuscular dynamics in ways that are not feasible experimentally. By bridging this gap between experimental data and modeling, this study has the potential to contribute to the development of more effective, personalized treatment strategies for age-related and neurodegenerative disease mechanisms of motor unit reorganization, and their interrelationships with muscle force production.

## Materials and Methods

### Overview

**Fig. 1:**
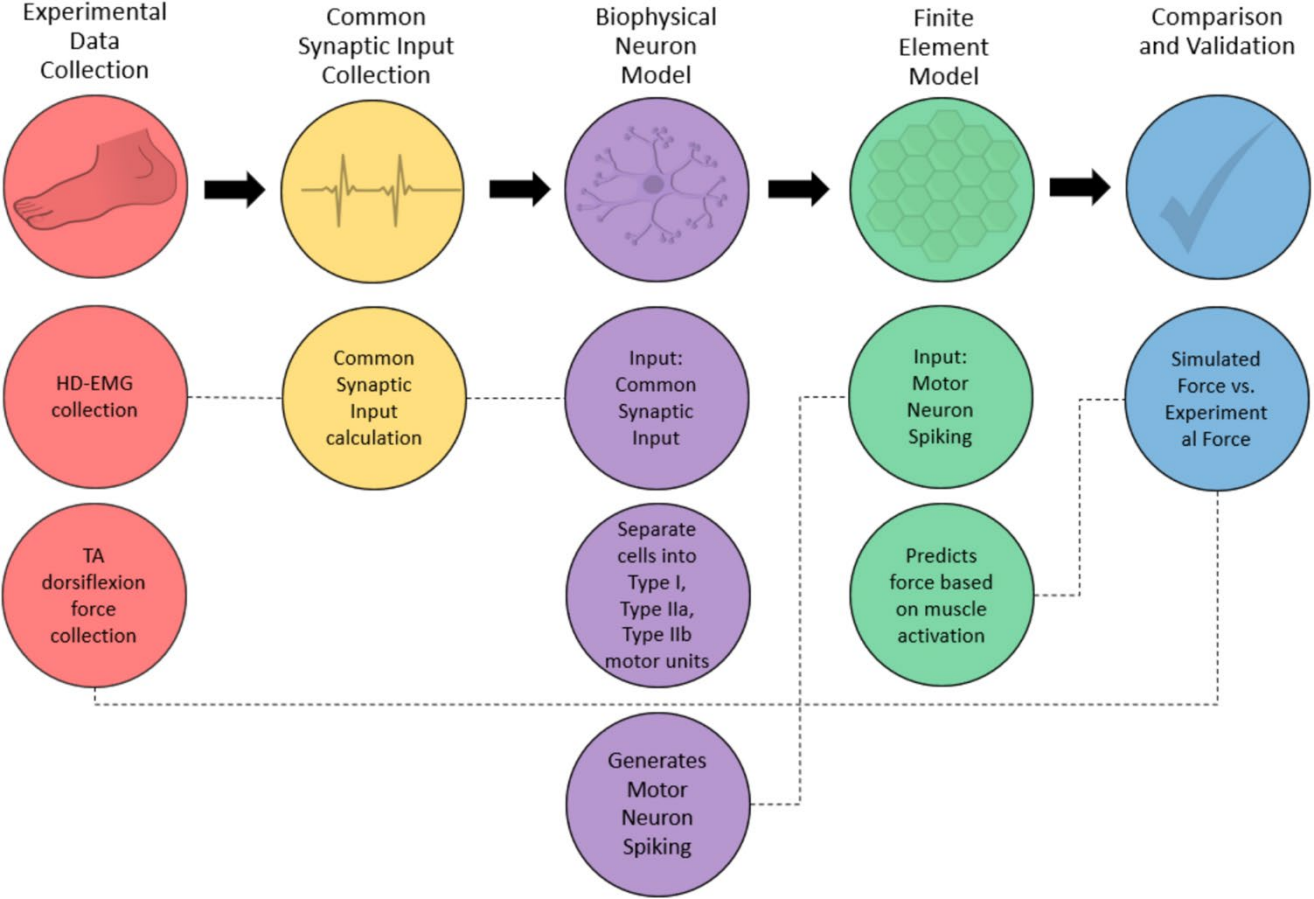
Workflow for neuromuscular modeling: From experimental data collection to validation, incorporating common synaptic input, biophysical neuron modeling, and finite element modeling to compare simulated and experimental force outputs

This research aims to develop a methodology for predicting subject-specific dorsiflexion force profiles by incorporating experimental MU recordings into a previously published FE musculoskeletal model (Fig. 1) [32]. This process serves to validate these NMS simulations through the comparison of CoV of ISI and STD of the CST, with experimentally collected MU data. We also compare STD and CoV of force as well as the profile of the resulting dorsiflexion simulation with experimental force recordings. These metrics were chosen as they have been used to compare subject’s dorsiflexion trials in previous research and were thought to represent fluctuations in shared and independent synaptic inputs to MUs [33–36]

### Participant Characteristics

Twenty-five college-age male participants (21.8 ± 1.7 years, 185.6 ± 7.5 cm, 83.12 ± 10.1 kg) were recruited to participate voluntarily in the study after written consent was granted. Participants did not have any lower limb injury or surgical procedure six months prior to the study. Additionally, individuals with history of repetitive lower limbs injury, neurological, or cardiovascular diseases were excluded from the study. Further, participants were physically active without being engaged in any specific sport or exercise program. Lastly, all experimental procedures adhered to the standards of Ethics Committee on Human Research of Aristotle University of Thessaloniki, in accordance with the Declaration of Helsinki (ERC-009/2022).

### Experimental Data Collection

#### Force Recordings

**Fig. 2:**
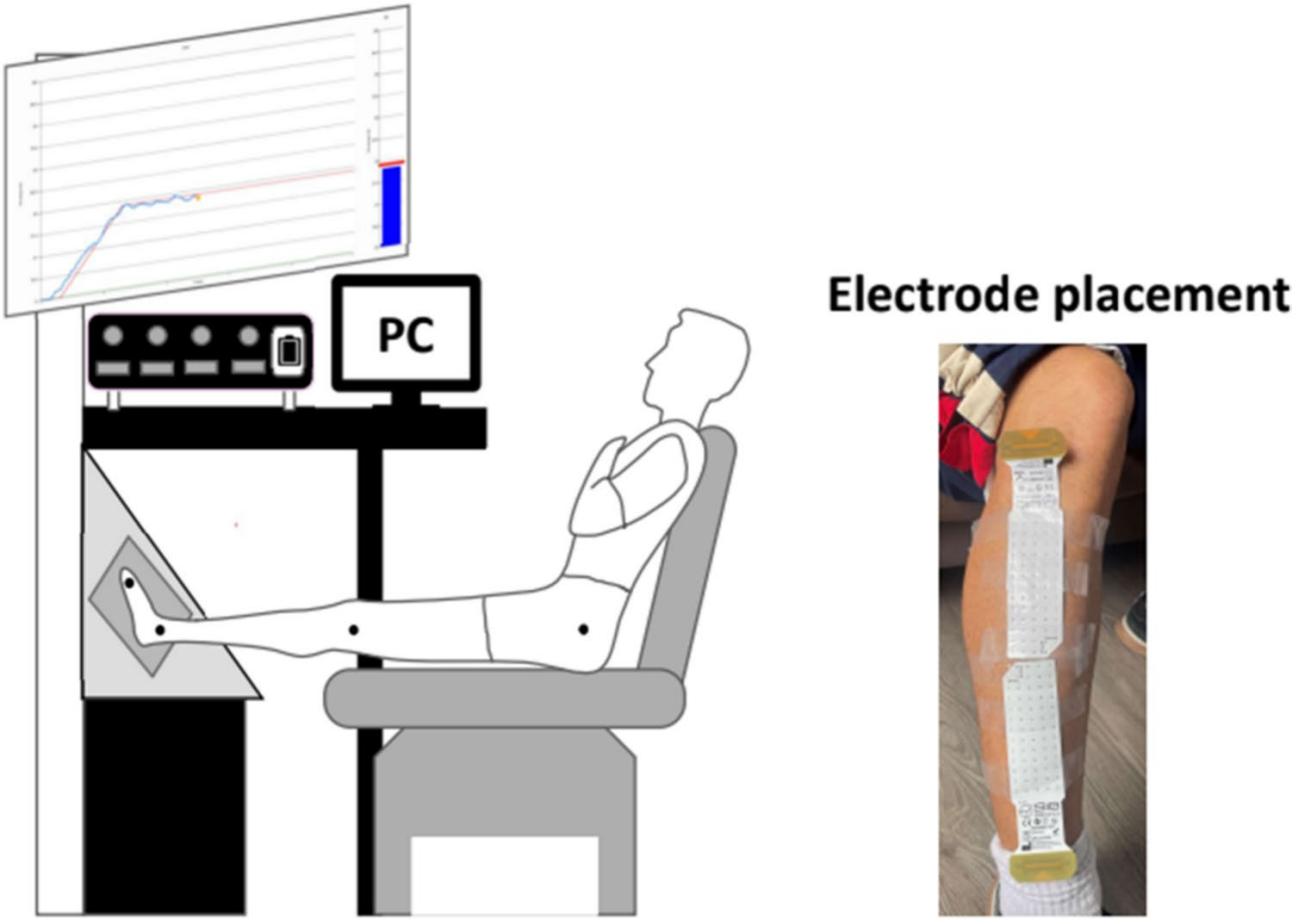
Experimental setup and electrode placement. Participants were seated in a reclined position with the foot secured to a dynamometer for isometric dorsiflexion force measurements. High-density surface EMG (HD-EMG) signals were recorded from the tibialis anterior (TA) muscle using a 64-channel electrode grid, shown on the right, positioned over the muscle belly

Participants performed maximal voluntary contractions (MVC) and trapezoidal contractions under isometric dorsiflexion conditions (Fig. 2). Maximal force was determined by two 5-s MVC trials where they were instructed to gradually increase the level of effort from rest to maximum and sustain this force for ~ 3 s. The highest MVC value between two trials was used throughout the experimental procedure to define the submaximal target forces. If the difference between the two trials was > 10%, additional trials were performed.

Trapezoidal ramp-and-hold isometric dorsiflexion contractions were performed in a randomized order between participants with visual feedback at 5, 10, 20, 40, and 60% MVC, and the rate increase/decrease of force set at 5% of MVC per second. The rate increase/decrease of 5% MVC is a slow steady rate that enable clear distinction of MUs recruitment and decruitment at the beginning and the end of the contraction. Duration of the isometric plateau was progressively reduced in order to avoid fatigue. At 5 and 10% of MVC the plateau duration was 30 s, while at 20, 40, and 60% it was 20-, 15- and 10 s, respectively [37, 38]. Participants performed three trials at each MVC force target with a rest period of approximately 1 min between trials. In order to ensure the rest period was sufficient and the effect of the previous trial was minimized, variability of the exerted force at the beginning and end of the plateau was confirmed to be similar and participants were required to report discomfort on a scale from 1 to 10, indicating extreme discomfort, with the average response being 2 ± 0.7.

#### EMG Recordings

Surface HD-EMG (HD-sEMG) signals were recorded with Bio Lab + software using two 64-channel grid electrodes (5 × 13, 8 mm interelectrode distance) placed over the proximal and distal portion of the tibialis anterior (TA). Semi-disposal foams were place on the surface of grid electrodes and holes were filled with conductive cream. Before placing the grids over the muscle belly the skin was shaved and rubbed with water to reduce skin’s impedance [39]. Ground electrodes were placed on the homonymous limb and right wrist using strap electrodes. Monopolar recordings of HD-sEMG were acquired, band-pass filtered between 20 and 500 Hz, using a second-order zero-phase Butterworth filter, digitized with sampling frequency of 2048 Hz on Quattrocento, 400 channel EMG amplifier, (OT Bioelettronica, Italy) and saved in hard disk.

HD-sEMG signals were decomposed offline using the semi-automated Convolution Kernel Compensation (CKC) algorithm, implemented in the DEMUSE tool software (version 5.01; The University of Maribor, Slovenia), to identify MU discharge times from each grid electrode. This method has been validated previously on identifying MU discharge times from HD-sEMG signals [40, 41]. Furthermore, it is proven that this method is able to identify MU discharge times with sensitivity > 90% and falsely identified discharges < 2%, for the target forces that were used during our experimental protocol [42]. Subsequently, identified spike trains from individual MUs were further visually inspected and manually edited, in order to correct any misidentified discharges. Discharge times variability and neural drive variability were only calculated from spike trains with pulse-to-noise ratio (PNR) greater than 29 dB, in order to ensure the quality of the decomposition results. Similarly, MUs with very short ISI (ISI < 20 ms) or high discharge times variability were discarded (ISI > 250 ms) [43]. Muscle activation recorded during participant dorsiflexion was quantified by the average root mean square (RMS) for each HD-sEMG grid electrode during the 6 steadiest seconds. Grid electrodes were separated vertically to apply single differential spatial filter, band-pass filtered (20–500 Hz), and summed to calculate the average value for each grid electrode.

Of the 25 participants in experimental data collection, the number included for computational NMS model development was reduced to 13 after imposing selection criteria based upon the effective capture of a participant’s neural recordings. We selected the best trial at the 60% MVC level based on three criteria: (1) a symmetric force profile that closely matched the visual feedback, (2) the highest number of recorded motor units (minimum threshold of 11 motor units), and (3) the most consistent firing of the motor units without experimental recording artifacts. This 60% MVC force target provided the best experimental condition to record changes in concurrently discharging action potentials and ensured that the model was driven by the most reliably recorded neural data, improving the accuracy of musculoskeletal force predictions.

### Computational NMS Model Development

**Fig. 3:**
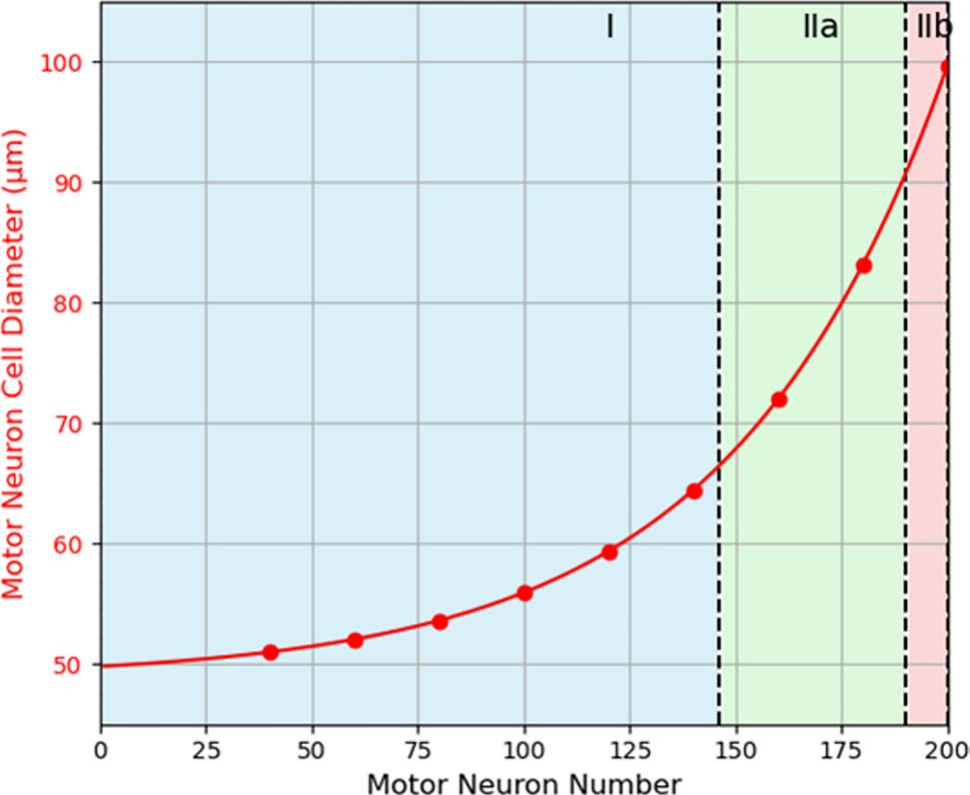
Relationship between motor neuron number and cell diameter: smaller motor neurons correspond to Type I fibers, while larger motor neurons innervate Type IIa and IIb fibers, reflecting the size principle of motor unit recruitment

We based our NMS model on a previously published biophysical model [20]. In brief, we integrated a previously established MU NEURON model [44] with the Abaqus/Explicit (SIMULIA, RI) FE software. The NEURON model, originally developed in the NEURON simulation environment, included detailed simulations of 311 dendrites, a soma, and axonal structures, incorporating potassium, sodium, and calcium channels to capture spiking and bursting activities essential for muscle force generation [44]. In the updated model, we expanded the motor neuron pool to include 200 neurons, with varied soma diameters so that recruitment dynamics could be accounted for in accordance to the Henneman size principle (Fig. 3) [45]. Motor neuron diameter was defined in a physiologically representative range (48.8–99.7 μm) for motor unit recruitment to occur following an exponential distribution. The peak twitch force for each motor neuron was calculated using an exponential distribution with a 100-fold range. In our model, the mapping between experimentally identified motor units and the modeled motor neuron pool is not a one to one. We applied the 100-fold exponential distribution to assign soma diameter and model fiber type in accordance with the size principle and physiological ranges.

The 3D FE model of the shank and TA muscle was constructed using cryo-slice images from the male Visible Human dataset [46, 47] and meshed with triangular surface (bony geometry) and tetrahedron elements for the muscle and tendon. The TA muscle’s material properties were modeled using a well-established framework, including active and passive muscle properties and fiber-specific muscle activation levels calibrated to experimental data [48]. This material model allowed for input of muscle activation levels and preferred direction of contraction or stretch for each element (related to the fiber direction of the muscle) into the model. The material model based on Lu [30] relates muscle activation levels, with values ranging from 0 to 1 that are generated via calcium dynamics which are determined by N-type and L-type calcium channels within the motor neuron model, to stress in each element of a continuum framework. Each element is assigned a specific fiber type, based on percentage distribution of fibers throughout the TA, with stress in each fiber type generated at a specific activation level. The material model, based on the Hill three-element model, consists of a contractile element and two elastic elements: one in parallel and one in series. The calculated stress generates a change in length for each element along its preferred contraction direction (i.e., it is assigned fiber alignment) which causes contraction of the muscle as a whole. A sensor, in the form of a constrained axial connector between the end of the TA distal tendon and the midfoot, was used to track the overall force generated by the TA during the dorsiflexion activity.

**Fig. 4:**
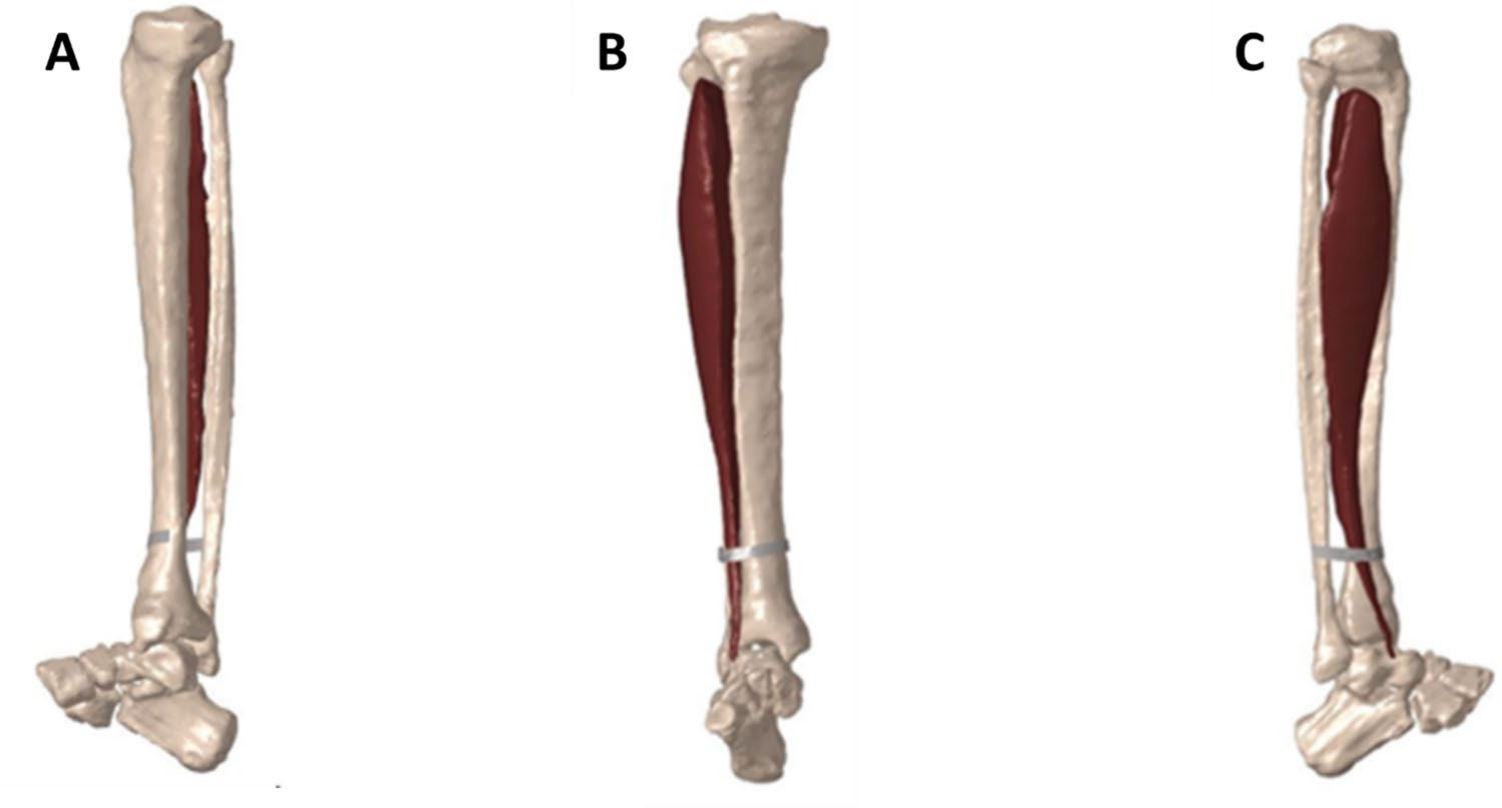
FE lower limb model of TA and surrounding bone geometry. Lateral (**A**), anterior (**B**), and medial (**C**) view of the right shank

The FE model does not consider individual fibers or assign twitch force but focuses on bulk muscle behavior. While the model does consider differential activation dynamics across fiber types (e.g., type I vs. type II), the mechanical dynamics are captured through a continuum-based material model that reflects passive and active properties of muscle tissue but does not explicitly model single twitch responses. The FE component translates a temporally evolving bulk activation signal into spatially distributed force generation across the muscle geometry. The motor neurons, based on soma diameter, innervate the elements in the FE model, which contract in directions corresponding to the bulk muscle fiber direction. The spiking recorded by the motor neurons are converted to “muscle activation levels” which is a relationship that describes how voltage signal from the motor pool activates calcium release in the sarcoplasmic reticulum at the muscular level and the resulting force from that calcium release. These muscle activation levels input in the FE model which then corresponds to deformation (contraction) of the elements in the muscle. For the tibialis anterior muscle, the fiber distribution is approximately 73% type I, 21% type IIa, and 6% type IIb fibers. Each motor neuron group controls a specific pool of fibers, consistent with the typical fiber composition in this muscle. This integration allowed for detailed simulation of TA muscle function, including force generation during dorsiflexion, aligned with in vivo characteristics (Fig. 4) [32].

**Fig. 5:**
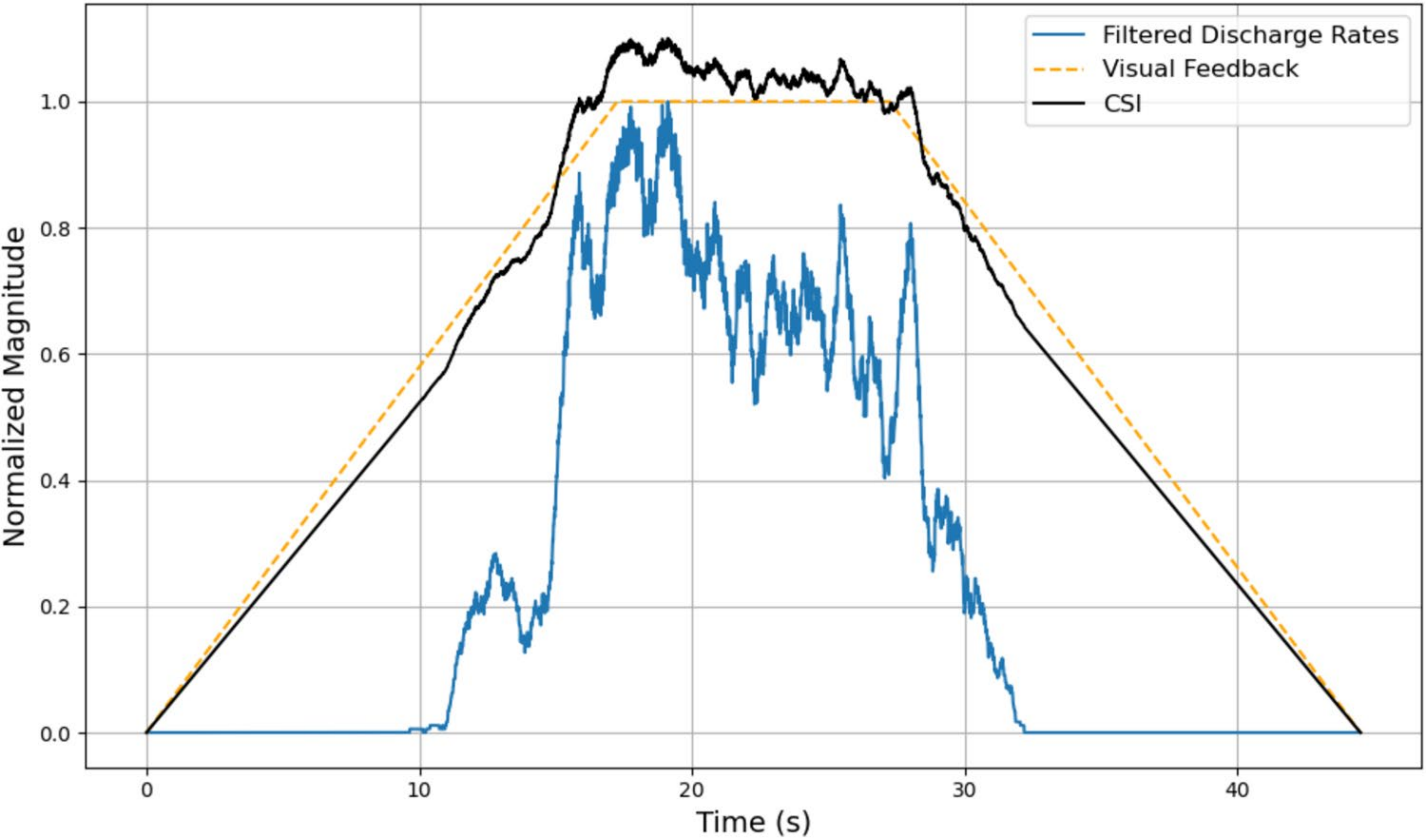
Representation of the two signals in which a weighted average is performed to generate the CSI for the neuromuscular model. The blue curve is a filtered version of the discharge rates of the participant’s specific HD-sEMG recording. The orange curve represents the desired force profile the subject is attempting to recreate. Finally, the black curve is the product of the weighted average of the orange (90%) and blue curve (20%) which is the CSI that is input into the motor neuron pool

The common synaptic input (CSI) represents the shared signal experienced by all motor neurons (MN) within a given pool. The CSI is comparable in shape to the discharge rates of the CST, the combination of all motor unit spike trains, of the experimentally collected data [12]. Therefore, the base shape of the modeled CSI was determined by the discharge rates of the experimental data. The shape of CSI was generated as a weighted average of the trapezoidal ramp-and-hold desired force profile (i.e., the visual feedback given to the participants) and the low-pass finite impulse response filter of the discharge rate of the CST with weightings of 90 and 20%, respectively (Fig. 5). These weighting were calibrated to best fit the experimental force profile. The experimental MUs used as input are not assigned specific fiber-type labels; instead, they inform the shape of the common synaptic input, which drives recruitment within the model based on motor neuron size. However, uncorrelated inputs to MNs, which is the input current that is unique to each motor neuron, also contributes to the total input current of the individual motor neuron. Individual motor neuron noise was modeled by adding random Gaussian noise within the range of − 1nA to + 1nA to the individual CSI which is the input to each individual motor neuron [14]. STD of CST was calculated by combining all motor units in simulated motor pool and experimental motor pools and then analyzing discharge rates (i.e., the number of action potentials of the entire pool in a given window) of the CST in 500-ms windows. The STD is then taken for each window as well as the ISI calculation.

Simulated MU recordings from each subject in the dataset were used as input to our previously published NMS model [32] so that subject-specific dorsiflexion force profiles could be predicted. The musculoskeletal FE model in this study was used to determine whether incorporating a CSI based upon experimental recordings could accurately predict experimental force fluctuation during isometric contraction. During isometric contraction, it is well documented that there is a degree of force fluctuation around the target force output [49]. In this NMS model, independent inputs to MNs, thus uncorrelated, have minimal impact on overall force production. Uncorrelated inputs are effectively canceled out at higher activity levels [50]. We performed a sensitivity analysis where biophysical parameters were perturbed across the motor neuron pool and noise artificially injected to individual motor neurons., The force variability was primarily influenced by the group behavior (common signal) of the motor neuron pool with individual MN noise not significantly affecting force variability., Force profile was primarily governed by the common synaptic input, estimated from the experimentally derived CST of concurrently discharging motor unit activity, which aligns with physiological principles. To analytically compare the model-predicted force output to the experimentally recorded subject-specific force output, we compared the CoV and STD of the two force profiles. The MVC level that was simulated and compared against the experimental trials was the 60% MVC level. These two metrics were calculated from the steadiest 6-second interval of the isometric contraction of the experimental profile and the steadiest 1-second interval of the simulated profile as the simulated profile was scaled in time in order to increase computational efficiency [33]. To further explore the differences between the simulated and experimental data, the STD of the CST was transformed into z-scores, and a correlation analysis was performed on the medians of these z-scores of both simulated and experimental conditions. We also compared CoV of the ISI for both experimental and simulated spike trains and performed a correlation analysis for each 500-ms window.

To evaluate the accuracy of our NMS model in predicting force output, we used two common performance metrics: the root mean square error (RMSE) and the coefficient of determination (*R*^2^). The RMSE quantifies the average magnitude of error between the experimental and simulated force data, providing a measure of absolute error in the same units as the force data (Newtons). Lower RMSE values indicate a smaller overall deviation between simulated and experimental force profiles. The coefficient of determination (*R*^2^) assesses the proportion of variance in the experimental data that is explained by the simulated data. An *R*^2^ value close to 1 indicates strong agreement between the experimental and simulated force profiles, whereas lower values indicate greater discrepancies. These metrics were computed for each trial and averaged across subjects to quantify the overall performance of the model.

### Sensitivity to Physiological Parameters

A sensitivity analysis of the physiological parameters of the motor neuron pool was conducted to evaluate the effect of NEURON model parameter selection on MU and muscle predictions. The sensitivity analysis examined the impact of varying 37 parameters on our NMS model (Table 1), with a focus on parameters, such as ion channel conductance at different physiological locations (soma and dendrites). For example, the conductance of the soma’s active ion channels and the passive ion channels were perturbed to show how the variation in these parameters affect the discharge characteristics of the motor neurons within our model. Each parameter was varied by ± 20% in 5% increments. Outcomes as previously described in this study (i.e., CoV of ISI, STD of CST) were used to analyze individual physiological parameters associated with motor neurons within a range of ± 20% of the original NEURON model to identify sensitive and insensitive modeling parameters as well as demonstrate the flexibility of the NEURON model to simulate different pathologies at the 60% MVC level.

**Table 1:** Parameter location and description for parametric study

Feature	Parameter
**Dendrite**	Membrane capacitance
	**Reversal potential of leak channels**
	**Peak conductance of leak channels**
**Hillock**	Membrane capacitance
	Reversal potential of leak channels
	Reversal potential of potassium channels
	Reversal potential of sodium channels
	Peak conductance of leak channels
	Peak conductance of potassium delayed rectifier channels
	Peak conductance of persistent sodium channels
**Initial segment**	Membrane capacitance
	Reversal potential of leak channels
	Reversal potential of potassium channels
	Reversal potential of sodium channels
	Peak conductance of potassium delayed rectifier channels
	Peak conductance of fast sodium channels
	Peak conductance of persistent sodium channels
**Soma**	Membrane capacitance
	**Reversal potential of leak channels**
	**Reversal potential of potassium channels**
	**Reversal potential of sodium channels**
	**Peak conductance of leak channels**
	Peak conductance of n-type calcium current
	**Peak conductance of calcium-dependent potassium channels**
	**Peak conductance of potassium delayed rectifier channels**
	Peak conductance of fast sodium channels
**NMJ**	Rate constant determining calcium concentration in the sarcoplasm
	**Release of calcium (rate)**
	**Time constant used to determine reuptake**
	**Reuptake of calcium (rate)**

Parameters in bold were found to influence model outputs in a sensitivity study.

## Results

**Fig. 6:**
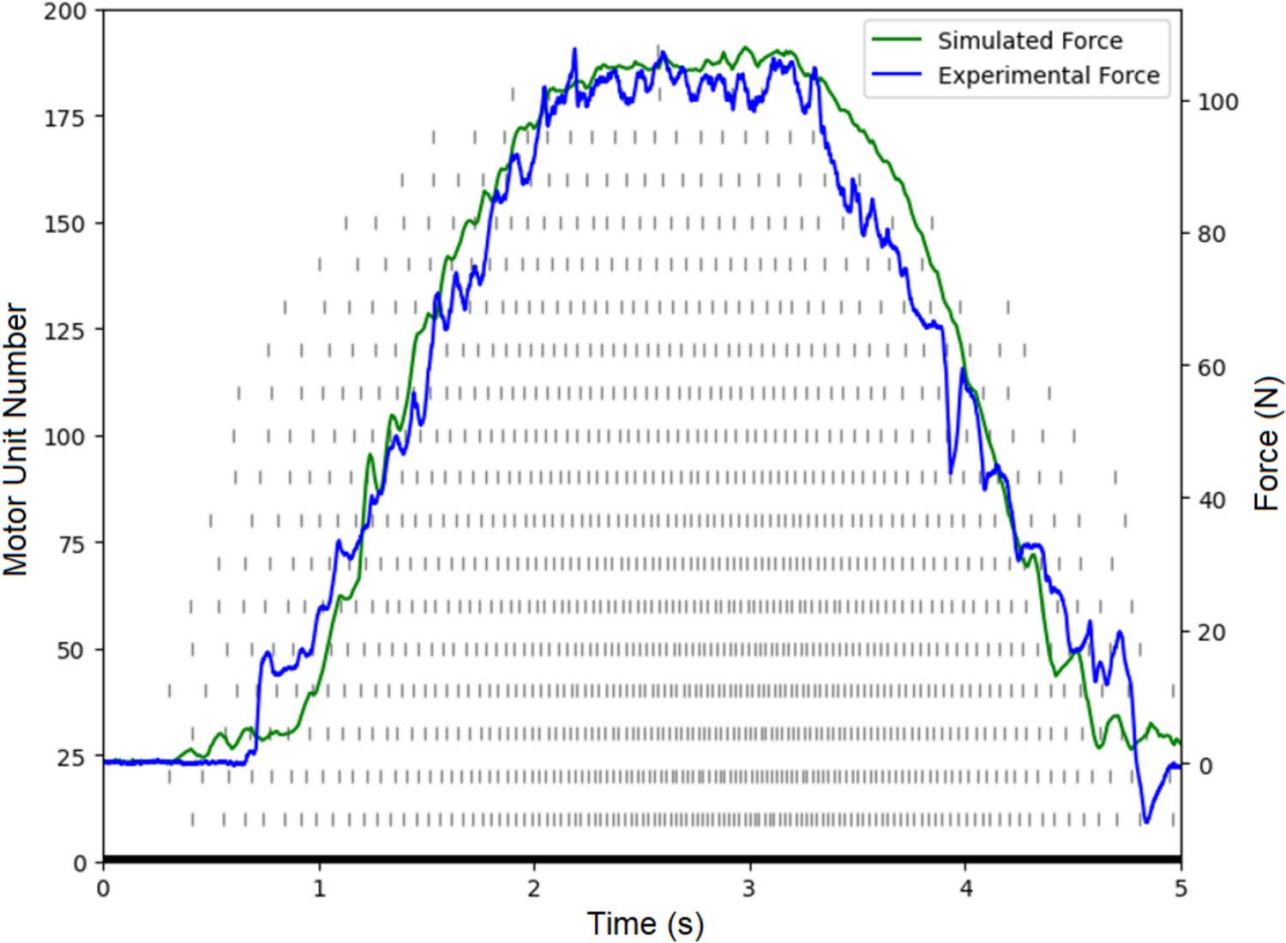
Force profile of simulated dorsiflexion (green) and experimental dorsiflexion (blue) with simulated raster plot (gray) with subject-specific force metrics

The average experimental force exhibited a STD of 1.32 and a CoV of 1.24%, while the simulated force exhibited a lower STD of 0.93 with a CoV of 0.91%. These values suggest a slightly higher variability in the experimental data compared to the simulations (Fig. 6). A statistical comparison of the standard deviations between the simulated and experimental data using a *t* test resulted in a t-statistic of − 1.421 with a *p* value of 0.090, indicating a trend toward statistical significance, though not conclusive. The *t* test for the coefficient of variation of force yielded a *t*-statistic of − 1.263 and a *p* value of 0.115, suggesting no significant difference between simulated and experimental CoVs of force.

**Fig. 7:**
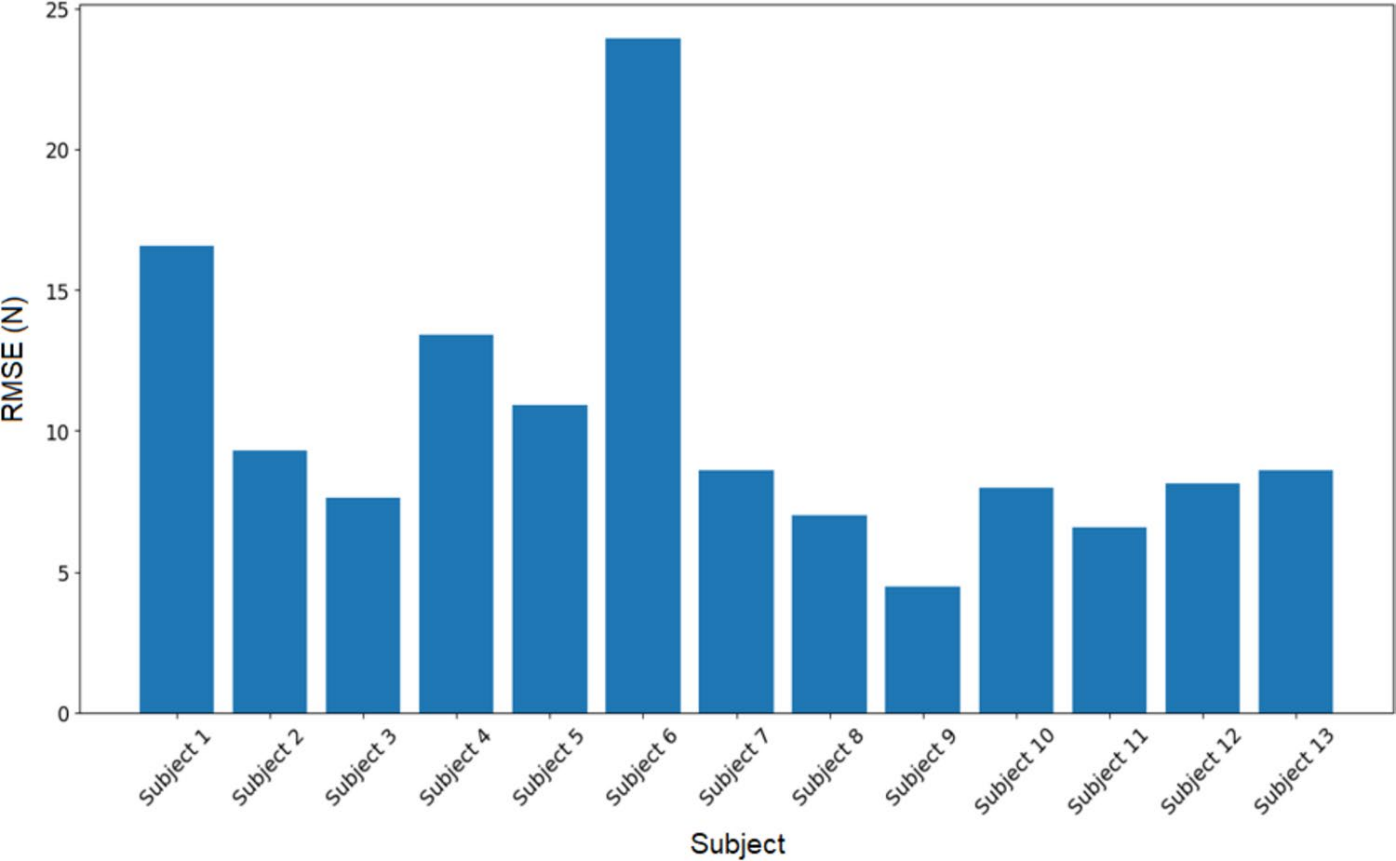
RMS error between simulated and experimental force profiles, illustrating the force differences between experiment and simulated predictions

The accuracy of the simulated force profiles in predicting the experimental force was further evaluated using the average root mean square error (RMSE) and *R*^2^ values, which were 10.25 N and 0.95, respectively. These results demonstrate a strong agreement between the simulated and experimental force profiles (Fig. 7).

**Fig. 8:**
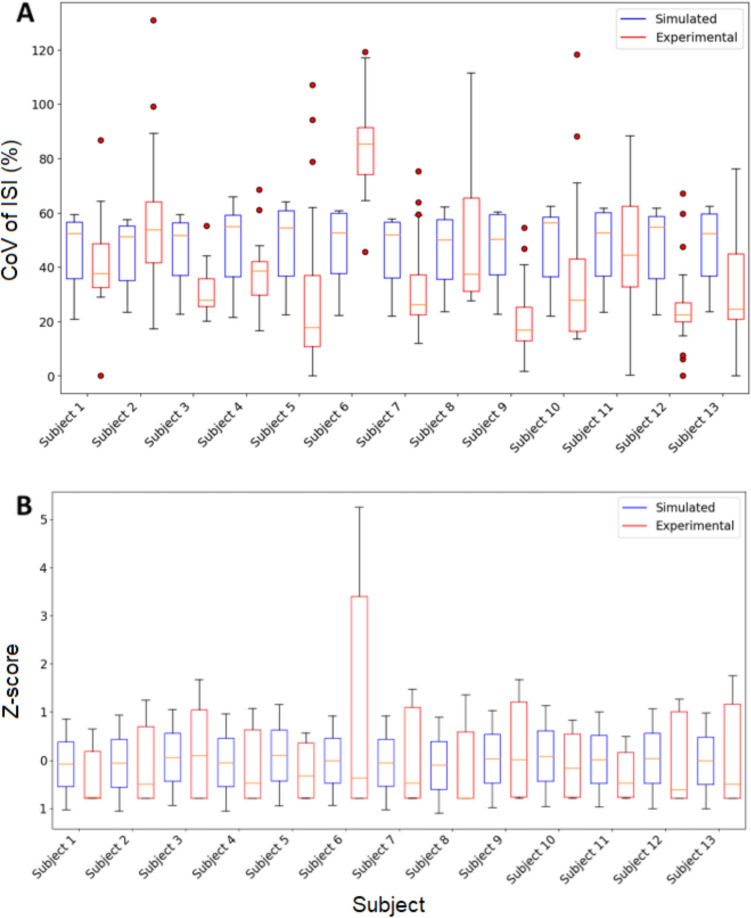
Boxplots of spike train metrics for each subject showing CoV of ISI (**A**) and Z-score boxplot of STDs of CST (**B**). The boxes show the middle 50% of the data with median line, with the whiskers indicating 1.5 times the interquartile range from first quartile and the third quartile (outliers are data points outside this range and indicated with red dots)

To further explore the differences between the simulated and experimental data, the STD of the CST was transformed into z-scores, and a correlation analysis was performed on the medians of these z-scores. The correlation coefficient between the simulated and experimental STD was found to be 0.71, with a statistically significant *p* value of 0.0062, indicating a strong positive relationship. In contrast, the correlation coefficient between the simulated and experimental CoV of the ISI was − 0.11, with a *p* value of 0.71, suggesting no significant relationship between these two measures (Fig. 8).

Of the 37 parameters tested, 16 were found to significantly affect the model’s performance (Table 1). We observed changes in discharge rates and muscle activation levels, or modeled muscle force production, in response to parameter variations. Parameters related to ion channels within the soma and dendrites had notable effects on discharge rates, while changes at the neuromuscular junction (NMJ) influenced muscle activation levels. All simulations were performed at the 60% MVC level to be continuous with the previous simulated and experimental comparison previously discussed.

**Fig. 9:**
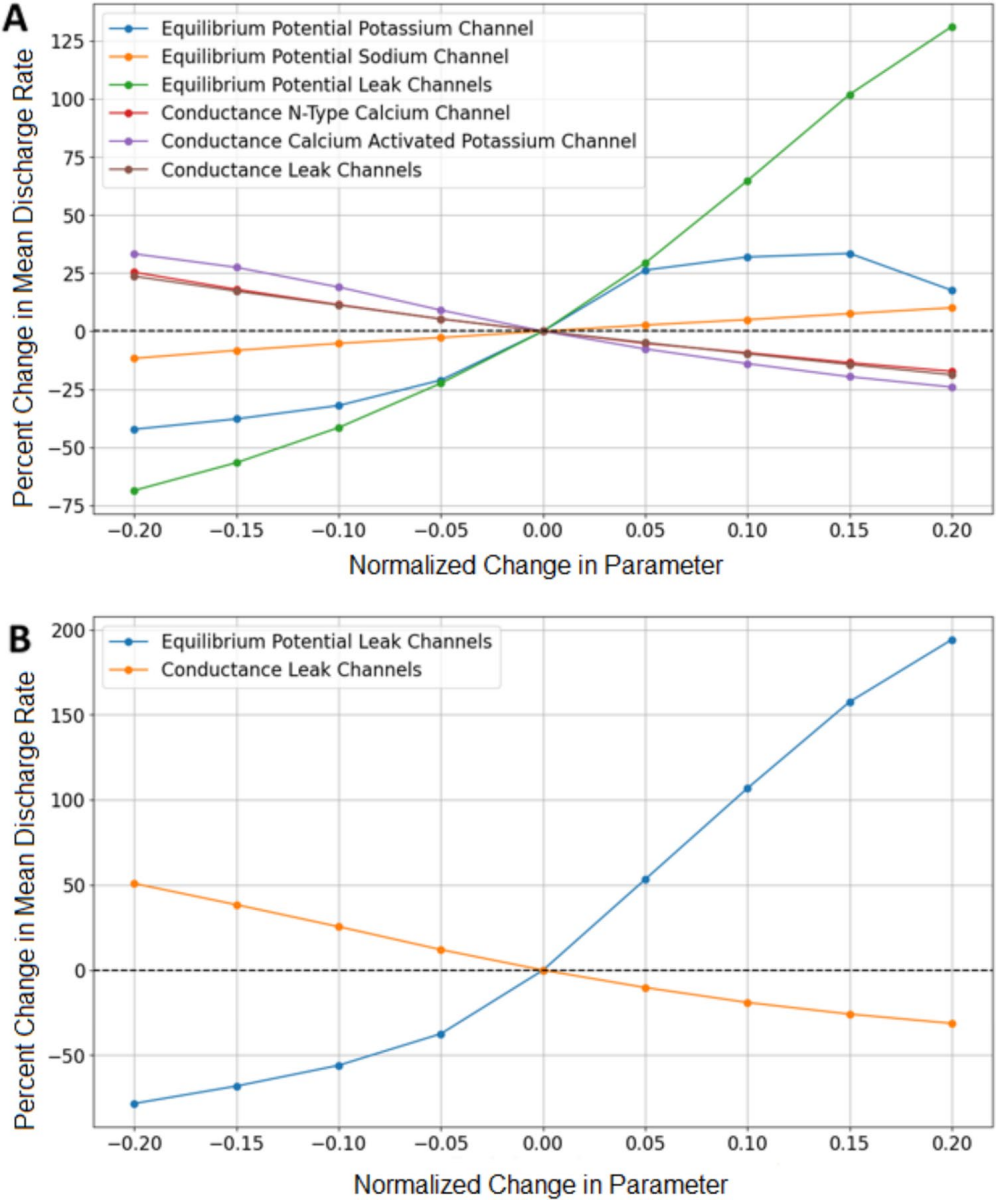
Sensitivity study results. Change in discharge rate vs normalized change in parameter associated with soma (**A**). Change in discharge rate vs percent change in parameter associated with dendrite (**B**)

The equilibrium potential of passive channels within the soma produced the most significant effects on discharge rates. Variations in this parameter resulted in a change in discharge rates ranging from a decrease of 68.59% at − 20% to an increase in discharge rates of 131.21% at + 20%. Other parameters showed a smaller range of effects of approximately 30% increase in discharge rates to a decrease 45%—there was a negative correlation in conductance of all ion channels and discharge rates as well as the equilibrium potential of the potassium channel. The equilibrium potential of the sodium channel in the soma was positively correlated with discharge rates. Similarly, the equilibrium potential of passive channels in the dendrites led to a substantial positive correlation with the discharge rates, with variations ranging from a decrease of 78.33% at − 20% to an increase in discharge rates of 194.14% at + 20%. This underscores the critical role of dendritic ion channel properties in modulating neuronal activity (Fig. 9).

**Fig. 10:**
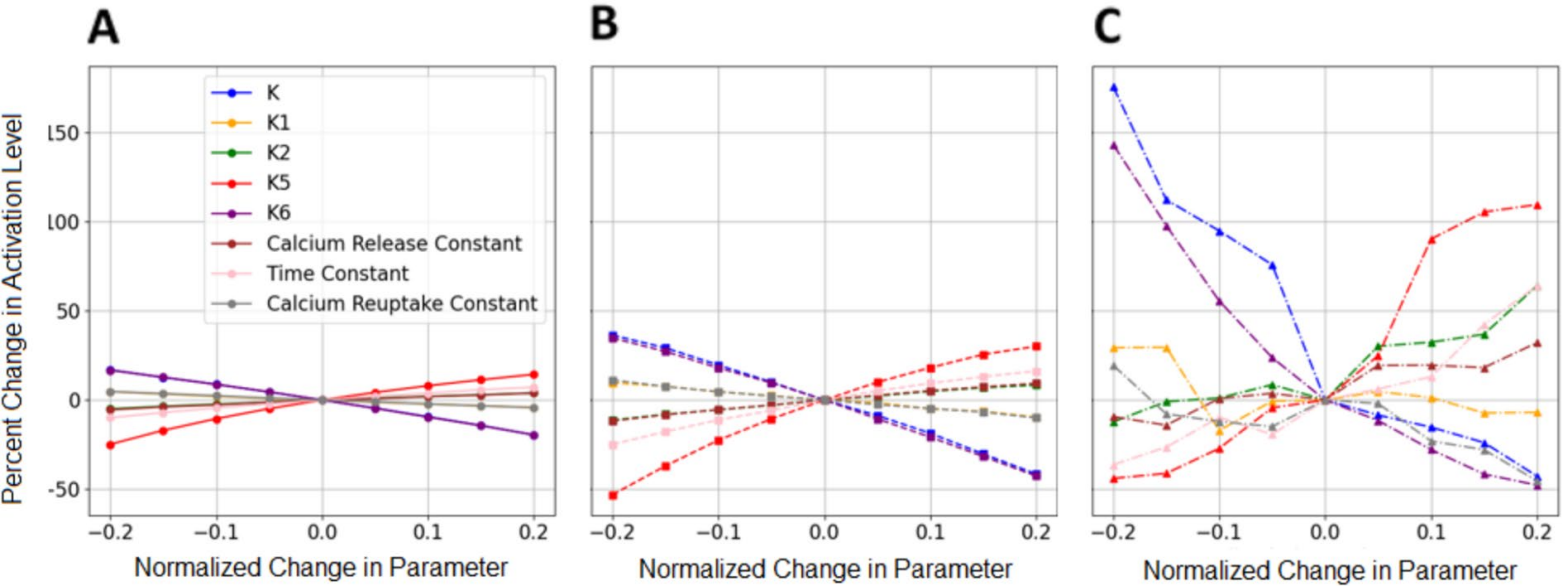
Change in activation level vs normalized change in a parameter associated with the NMJ activating type I (**A**), type IIa (**B**), and type IIb (**C**) muscle fibers

At the NMJ, the parameter ***k***, which represents a rate constant determining calcium concentration in the sarcoplasmic reticulum (***k***_***1***_***, k***_***2***_) and reuptake of calcium back into the sarcoplasm (***k***_***5***_***, k***_***6***_), exhibited the most pronounced effect on muscle activation levels. Variations in ***k*** led to substantial changes in activation highlighting the sensitivity of muscle fiber activation to changes in calcium handling dynamics. For type I and type IIa muscle fibers, activation levels varied linearly with parameter changes. These changes for type I fibers ranged from an increase in discharge rates by 25% to a decrease in discharge rates of 25% with an increase in discharge rates for type IIa fibers of 40% to a decrease of 52%. In contrast, type IIb muscle fibers demonstrated a higher sensitivity to parameter changes, likely due to a very small population of motor neurons innervating these fibers in comparison to type I and type IIa. The differing sensitivity among muscle fiber type and innervation ratio highlights the effects of individual motor neuron noise compared to the effect of the CSI. The activation levels for type IIb fiber vary from an increase of 175.5% at − 20% to a decrease in discharge rates of 42.7% at + 20% (Fig. 10).

## Discussion

The high *R*^2^ value (0.95) and low RMSE (10.25 N), which is within the reasonable range of variability demonstrated by participants’ own ability to match real-time visual feedback in experiments of a similar setup (e.g., 7.15 N with visual feedback), obtained in our simulations suggest that the NMS model accurately predicts dorsiflexion force profiles based on experimental data. This level of accuracy indicates that our NMS model can closely replicate the force profiles observed in vivo, highlighting its potential utility in clinical scenarios. However, while the simulated data closely aligned with experimental outcomes, a trend toward higher variability in experimental data compared to simulations was observed. This discrepancy, with experimental force profiles exhibiting a higher STD (1.32) and CoV (1.24%) than simulated profiles (0.93 and 0.91%, respectively), may be attributed to inherent biological variability or simplifications in the NMS model such as not including single motor neuron to muscle fiber bundle control.

The spike train analysis further supports the effectiveness of the NEURON model in capturing neuronal activity. The significant correlation (*r* = 0.71, *p* = 0.0062) between simulated and experimental STD of CST indicates that the NEURON model adequately reflects the variability in neural drive delivered to the muscle which is associated with force generation. However, the lack of significant correlation (*r* = − 0.11, *p* = 0.72) between the CoV of ISI highlights a limitation in replicating the discharge times variability patterns observed in vivo. This suggests that while the NEURON model can simulate overall neuronal activity, it may not fully capture the intricacies of ISI variability which is most likely due to the physiological simplification of this model. During an analysis of MU decoded and the resulting force variability it was found that total number of MU’s decoded had a correlation with the force variability. A lower number of decoded MU’s was found to produce less force variability. While this is a limitation of the current model, in the current study, this is minimized due to the initial exclusion criteria of participant force trials. While our NEURON model is capable of simulating neuronal activity of MNs population as STD of CST, it needs further improvement to capture the independent variability of individual MNs. Inclusion of finer anatomical elements such as upper motor neurons (pyramidal cells) and the inclusion of multiple inputs to the motor neuron (sensory neurons, upper motor neurons, and interneurons) in these simulations may result in more representative ISI variability, as ISI is likely dominated by independent synaptic inputs.

Neurodegenerative diseases like Alzheimer’s, Parkinson’s, and ALS present significant challenges in healthcare, both in terms of prevalence and the profound impact on quality of life [4]. With ALS projected to increase 34% by 2040 and Alzheimer’s disease expected to double by 2060, there is an urgent need for innovative approaches to treatment and rehabilitation [4]. Traditional clinical trials, while essential, face ethical and logistical constraints, particularly in the context of neurodegenerative diseases where invasive procedures are often required. In this context, computational models offer a promising future alternative. In this study, we have developed and validated an NMS model that integrates complex neuronal components with FE capabilities to simulate subject-specific force profiles and neuronal activity. While this study focuses only on healthy participants, in future we envision this same approach applied to develop and validate neurodegenerative NMS models.

Current neuromuscular research is limited by inadequate knowledge of disease mechanisms, and the need for invasive procedures in clinical trials. Computational models can offer an efficient and non-invasive platform that could further the understanding of NMS conditions and research treatment options in a patient-specific environment [51]. Therefore, the development of fully predictive NMS models with the ability to integrate complex neuronal components with FE capabilities is necessary to simulate neurodegenerative and musculoskeletal conditions. These models should accurately simulate the interaction between the nervous system and musculoskeletal system to be effective in informing treatment for complex neurodegenerative diseases and predicting post-treatment function.

Our sensitivity analysis provides valuable insights into the impact of specific physiological parameters on the NEURON model performance. Notably, parameters related to ion channel dynamics, such as the equilibrium potential of passive channels in the soma and dendrites, significantly influenced discharge rates. The pronounced effect of the ***k*** parameter, related to calcium concentration at the NMJ, underscores the sensitivity of muscle activation levels to changes in calcium handling dynamics. These findings emphasize the importance of accurately representing key physiological factors in the NEURON model to ensure its reliability. The sensitivity results from this study demonstrate the potential of such models to provide a non-invasive, patient-specific platform for understanding and predicting outcomes in neurodegenerative diseases and musculoskeletal conditions.

With the development and increased usage of motor unit recording methods, such as HD-EMG, there has been an increase of experimental data which displays the discharge rate characteristics of patients with neurodegenerative diseases, such as Parkinson’s [52] and ALS [53, 54]. In future iterations of this NEURON model, this would allow us to compare our models results directly with experimental data. In ALS, voltage-gated sodium channels have been linked to hyperexcitability or a higher likelihood of producing an action potential, in the spinal motor neurons of SOD1^G127X^ mice [55]. In our model, this could be simulated by varying the conductance of sodium channels in the soma to induce hyperexcitability and increase discharge rates. Sodium channel dysfunction is also associated with hyperkalemic and hypokalemic periodic paralysis. In Anderson’s syndrome, a disease that causes muscle weakness and paralysis, the increase in potassium channel current leads to a decrease in motor unit discharge rate [56]. Malignant hyperthermia, an inherited condition that causes severe uncontrollable fever during the use of general anesthesia or muscle relaxants, is characterized by altered sarcoplasmic calcium release, resulting in sustained muscle contraction and fever [57]. This phenomenon can be modeled by varying the rate constant ***k***, which determines sarcoplasmic calcium release and reuptake. While this sensitivity study demonstrates the simple ability to effect discharge rates based upon the change of a single parameter, this illustrates that our NEURON model has the capability to represent multiple physiological parameters. In future work, we may calibrate the NEURON model parameters to better represent a specific neurodegenerative disease.

This foundational study combines neuromuscular and musculoskeletal simulation and underscores the ability of NMS models to simulate participant-specific neuromuscular and musculoskeletal parameters under healthy conditions. While our NMS model shows promise in simulating force profiles and neuronal activity, further refinement is necessary to fully capture the complexity and variability of the human neuromusculoskeletal systems under healthy and diseased states. Future work should focus on integrating additional biological factors that could influence MU discharge variability, such as muscle fatigability, shared and independent synaptic inputs delivered to MNs, and improved physiological and anatomical features such as pyramidal cells which are the start of the corticospinal tract, to enhance the model’s predictive power and clinical applicability.

The ability to simulate patient-specific conditions accurately could revolutionize treatment planning and outcome prediction in neurodegenerative diseases, providing a non-invasive, cost-effective tool that complements traditional clinical methods. As computational modeling continues to evolve, these models will increasingly help in bridging the gap between bench research and bedside care, offering new avenues for personalized medicine and improved patient outcomes.
